# Calibration of impulse high-voltage test systems above 500 kV peak: implementation and evaluation

**DOI:** 10.1038/s41598-026-50002-6

**Published:** 2026-05-04

**Authors:** Ahmed S. Haiba

**Affiliations:** Electrical Power, Energy and High Voltage Metrology Laboratory, National Institute of Standards (NIS), Giza, Egypt

**Keywords:** Calibration, Impulse high-voltage, Standard divider, Uncertainty, Validation, Energy science and technology, Engineering

## Abstract

The rapid technological advancements of today have ushered in a new era of impulse high-voltage testing equipment capable of reaching high voltage ranges. Traditional calibration methods fall short in calibrating such high voltage equipment. This study introduces an on-site calibration approach tailored for impulse high-voltage testing equipment with an 800 kV range. The method employs two standard dividers: one for measuring impulse voltage up to 500 kV peak and the other for DC charging voltage up to 100 kV. An efficiency factor technique is proposed for calibration beyond 500 kV peak. Results indicate achieved linearity across all calibrated points with a linearity error under 1%, validating the extension of calibration up to 800 kV peak. The unit under calibration aligns with specified accuracy standards. Uncertainty is evaluated for each range to ensure result reliability. A comparison and verification tool using a normalized error approach confirms the efficacy of the proposed technique, demonstrating satisfactory outcomes.

## Introduction

High voltage (HV) has several applications throughout industrial, medical, agricultural, and various other areas. Three categories of high voltage are common in nature: High Impulse Voltage, High Direct Current Voltage (HVDC), and High Alternating Current Voltage (HVAC)^1^. DC voltages are employed for the testing of HVDC equipment, HVAC items, and for research purposes^2,3^. High DC voltages are typically produced by the rectification of HVAC and other circuits^4,5^. HVAC generation may originate from Tesla coils, step-up transformers, cascade transformers, resonant transformers, etc. There are two categories of impulse voltages: lightning and switching impulse voltages^6^. Impulse voltages are employed for dielectric testing of high-voltage equipment, including power cables, power transformers, high-voltage insulators, and several other products. By definition, the impulse voltage is a purposefully created aperiodic transient voltage that rises quickly to its maximum value before gradually falling to zero. A lightning impulse (LI) has a front time of less than 20 μs, while a switching impulse (SI) is an impulse voltage with a front time longer than 20 μs^7,8^. To replicate the voltage stress brought on by a lightning or switching event in a supply network, test objects are subjected to impulse voltages. According to the relevant test standards, the amplitudes of these voltages during testing are often in the tens or hundreds of kilovolts.

IEC 60060-1:2025 states that there are three primary factors for impulse voltage: peak voltage Up, front time, or time to peak T1, and tail time, or time to half-value T2. T1 is the time interval between the start of the impulse and when it reaches its greatest value, T2 is the time interval between the start of the impulse and when the voltage first drops to half of its maximum value, and Up is the maximum value of the impulse voltage. T1 and T2 for the typical lightning/switching voltage are 1.2/50 μs and 250/2500 μs, respectively^8^. Advances in high-voltage generation have been made possible by researchers from all across the world^9–13^. High voltage items must be tested to make sure they meet the standards of the tests carried out in accordance with the applicable regulations. As a result, in order to ensure accuracy, high voltage testing equipment must undergo regular calibrations. Testing and instrumentation applications in the electric power industry require accurate high voltage measurements^14–18^. Therefore, many public and private organizations demand periodic calibration of high voltage measuring and sourcing systems to provide accurate electrical standards and allow for trusted measurements^19^. National metrology institutions in a number of nations have developed impulse high voltage standard measurement systems by means of component traceability techniques and international comparison of impulse voltage measurement equipment^20–23^.

Impulse voltages are often measured using a system that includes a digitizer (transient recorder) with a potential attenuator, a signal wire, and a voltage divider with a potential damping resistor^7^. The purpose of the damping resistor is to reduce undesired impulse transients. The high-voltage impulse is transformed into a low-voltage impulse while retaining its shape using a voltage divider. The low-voltage signal is sent from the voltage divider to the digitizer’s input via the signal cable. If the signal is too strong for the digitizer input, a second attenuation may be used.

According to IEC 60060-2:2025^24^, an acceptable impulse voltage measuring system must be calibrated on a regular basis and have an expanded uncertainty of less than 10% for the time parameters and less than 3% for the peak voltage value. For high-voltage equipment calibration procedures, a traceable reference standard is required to ensure the accuracy and performance of the equipment being calibrated. As recommended by the standard IEC 60060^8,24^, it is preferable to perform the calibration on-site by comparing it to a reference measuring system because the high voltage equipment is large and difficult to be transported.

High-voltage impulse test systems are usually calibrated at the customer’s site over a range up to 500 kV peak by National Metrological Institutes (NMI’s) as the National Institute of Standards (NIS) in Egypt. In fact, with the current technological boom, there are devices for testing impulse voltage above 500 kV that may reach 2400 kV or above, as is found in cable production factories in Egypt. Therefore, metrological institutes are limited to calibrating such devices up to 500 kV only and are unable to cover the range of devices exceeding this range at the customer’s site. Consequently, the reference standards present in national institutes are unable to cover the range of devices exceeding 500 kV. For this reason, this research paper discusses how to implement calibration of impulse high-voltage test systems that exceed the capabilities of metrological institutes.

In this study, an impulse high-voltage testing equipment up to 800 kV peak is calibrated using a 500 kV peak reference standard voltage divider. An on-site case study is used to illustrate the suggested approach. The proposed efficiency factor method is primarily applicable to peak voltage calibration for values exceeding the standard measurement range. In order to assess the efficiency factor of the test system being calibrated, the method depends on sensing the input DC voltage for charging during the calibration process. The linearity test is then applied to the measurements up to the measuring range of the standard divider. Based on this, the calibrated equipment can be reliably extended to its full impulse voltage range. Furthermore, the calibration process uncertainty budgets are determined and evaluated. Finally, the proposed methodology is investigated to ensure the accuracy and reliability of the results.

The main novelty of this work lies in the development of a practical on-site calibration methodology for impulse high-voltage systems exceeding the range of available reference standards. Unlike conventional methods that rely on full-range reference dividers or laboratory-based calibration, the proposed approach introduces an efficiency factor-based technique that enables reliable extension of calibration beyond 500 kV using a limited-range standard. In addition, the method integrates linearity analysis, uncertainty evaluation, and normalized error verification to ensure measurement reliability. This provides a practical and scalable solution for high-voltage calibration in real industrial environments.

## Experimental setup

### Calibration object

The calibration object in this study (UUC) is a lightning impulse voltage measuring system up to 800 kV peak with an accuracy of 3%. The measuring system under calibration consists of the following components:

- Damped capacitive impulse voltage divider of 1800 kV-peak with type of SMC 400/1800 and manufactured by HIGHVOLT company. This divider consists of two high voltage measuring capacitors with types of MWF 1000 kV–800 pF and MWF 800 kV–1000 pF. The later one is used for operation for measuring 800 kV lightning voltage.
- Low voltage measuring part of the divider with type of H391-14.
- Measuring cable (double screened) with 50 Ω and length 50 m.
- Digital recorder with type of MIA 200-12/2B for controlling and displaying the measured parameters through channel 1.

To ensure flexibility and produce the desired waveform, a multistage impulse generator circuit consisting of eight stages has been installed in addition to the calibration object. These elements fall under the following categories:

- DC Charging Unit: To fully charge the generator’s capacitors, this unit needs to supply a variable DC voltage with either positive or negative polarity.
- Charging Resistors: Usually ranging from 10 to 100 kilo-ohms, these are high-value, non-inductive resistors. Every resistor is designed to tolerate voltages ranging from 50 to 100 kV.
- Generator Capacitors and Spark Gaps: Designed for frequent cycles of charging and discharging, these capacitors are arranged vertically with all spark gaps aligned. In a short circuit, they can deliver currents of up to 10 kA. Although spherical-ended cylinders with central supports are occasionally employed, spark gaps are typically spherical or hemispherical, with a diameter of 10 to 25 cm.
- Wave-Shaping Resistors and Capacitors: These non-inductive, wound resistors have voltage ratings ranging from 50 to 100 kV per stage and can withstand impulse currents of up to 1000 A. The capacitances of load capacitors, which range from 1 to 10 nF, can be either oil-filled or gas-compressed.
- Triggering System: This consists of trigger spark gaps that are intended to start spark breakdown inside of them.

**Fig. 1:**
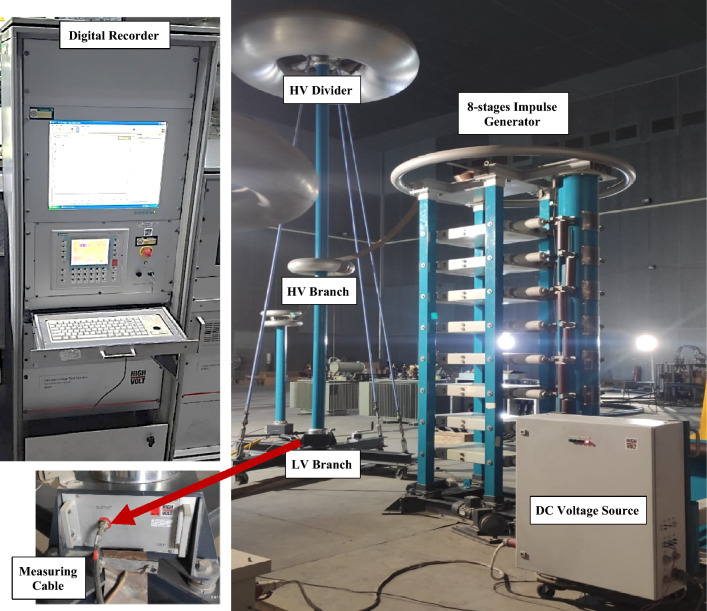
Impulse High voltage testing system under calibration.

The eight steps of the multistage impulse generator under investigation each have their own spark gap and capacitor. The output voltage is the total of the voltages of each individual step since the stages are connected in series. This makes it possible to generate extremely high voltages by cascading several stages up to a peak of 800 kV. The voltage divider measures this impulse output voltage. There are two branches on this voltage divider: high and low. The output of the voltage divider (low branch) is connected to the input of the digital recorder’s measuring device via a 50-m measuring cable. The digital recorder shows the voltage and time parameter measurement values. Figure 1 shows the unit under calibration of the measuring test system and the multistage impulse generator.

## Reference standards

**Fig. 2:**
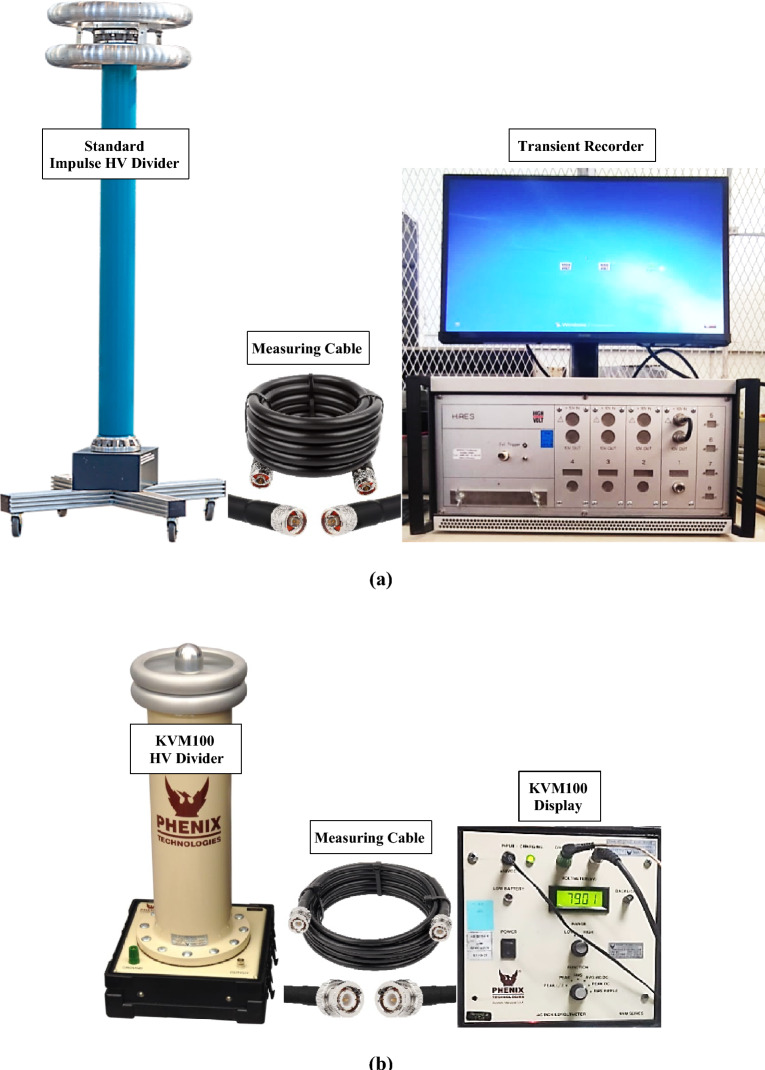
The used standard equipment: (**a**) Impulse high voltage measuring system, and (**b**) DC high voltage measuring system.

In this study, a standard of impulse high voltage measuring system is used. It consists of an impulse high voltage divider up to 500 kV peak value which is compatible with a transient recorder, HiRES, for displaying and recording the output measuring parameters such as peak voltages in kV and waveform times in μs via a measuring coaxial cable. The ratio of the divider is 500:1 V/V. This standard system has an accuracy of 1.0% and an expanded uncertainty of 0.65% for all ranges. In addition, a DC high voltage divider, PHENIX KVM100, is used for measuring the DC charging voltage generated from DC source and connected to its display via a compatible coaxial measuring cable. The DC voltage is measured during calibration process at each calibrated point. Figure 2 shows the standard equipment used in this study.

### Calibration procedure

As previously stated, the calibration of the high voltage equipment should ideally be done on-site by comparison with a reference measurement system because of the size of the equipment. The calibration is carried out in a customer laboratory setting. The high voltage side of the standard impulse divider is connected to the high side of the multistage impulse generator and the low voltage side is connected to the transient recorder HiRES via a coaxial measuring cable. By this step, the standard impulse measuring system is connected in parallel with the system under calibration. The KVM100 voltage divider is connected to the DC source and interlinked with its display via a measuring coaxial cable for measuring the DC charging voltage. By controlling the output impulse voltage, five calibrated points are taken in the measurement range of 500 kV at positive and negative polarities as 100 kV, 200 kV, 300 kV, 400 kV, and 500 kV. At each calibrated point, the standard measured value is compared with the corresponding value of the calibration object. Each point shall contain five readings at least at both polarities then an average value is obtained for each point. At the same time, the DC voltage shall be measured and recorded at each impulse voltage.

It has been seen that the range of the standard measuring system (500 kV) cannot cover the full range of the calibration object (800 kV). This means that the standard measuring system covers about 62.5% of the full range of the calibration object. According to IEC 60060-2^23^, If the standard measurement range is inadequate for covering the equipment under test, it should be set to a minimum of 20% of the highest voltage of the equipment under calibration. Therefore, statistical linearity tool has been introduced to provide an extension to the maximum voltage value of the calibration object. Consequently, the proposed approach is valid for the calibration of impulse voltage measuring systems up to 2500 kV. This ensures that the method is suitable for UHV applications.

This methodology depends on the efficiency factor estimation of the calibrated system to determine the standard value of the voltage over the range. The efficiency factor (η) is introduced to establish a relationship between the generated impulse voltage and the corresponding DC charging voltage. It is defined as the ratio of the measured impulse peak voltage to the product of the number of stages and the measured DC voltage, as given by Eq. (1).

1 $$\eta = \frac{{U_{imp} }}{{n \times U_{dc} }}$$

where ${U}_{imp}$ is the measured impulse peak voltage (kV); ${U}_{dc}$ is the measured DC charging voltage (kV); $n$ is the number of stages of the impulse generator.

The efficiency factor reflects the conversion efficiency of the impulse generator. It is calculated at each calibration point within the standard measurement range (up to 500 kV). When the variation of η remains within ± 1%, the average efficiency factor can be used to estimate impulse voltages beyond the standard range using Eq. (2).

2 $$U_{imp} = \eta \times n \times U_{dc}$$

A stable efficiency factor indicates a linear relationship between input and output voltages, enabling reliable extrapolation beyond the standard measurement range.

## Results and discussion

In this study, the calibration process is carried out through two steps. In the first step, the output impulse voltage is measured by the standard impulse high voltage measuring system up to its full range 500 kV peak and compared with that of the unit under calibration. At the same time, the DC charging voltage is measured by the standard DC high voltage measuring system, KVM100, at each calibrated point and recorded. In the second step, the standard impulse high voltage measuring system is disconnected and removed. Then, the output impulse voltage of the unit under calibration is applied and recorded up to the maximum full range of the testing system. In addition, the DC voltage is still measured at all calibration points. All measurements were carried out under environmental conditions with a temperature of (23 ± 2) °C and relative humidity of (50 ± 10) %.

## Calibration within the measurement range of the standard

**Fig. 3:**
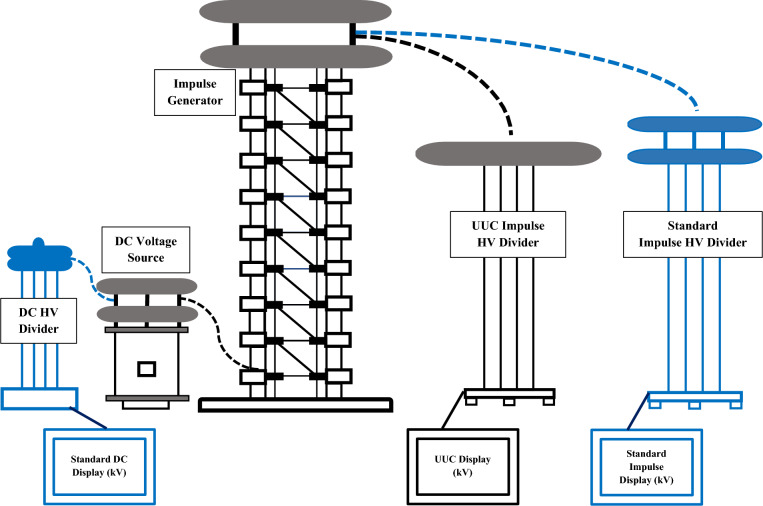
Connection diagram of the reference standards with the UUC.

**Fig. 4:**
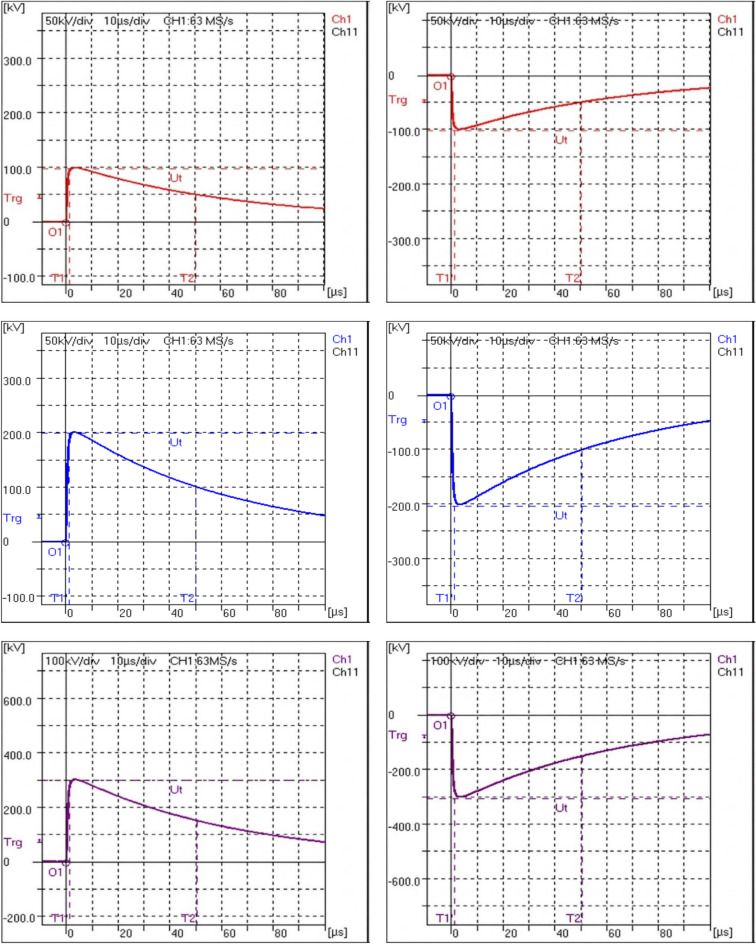

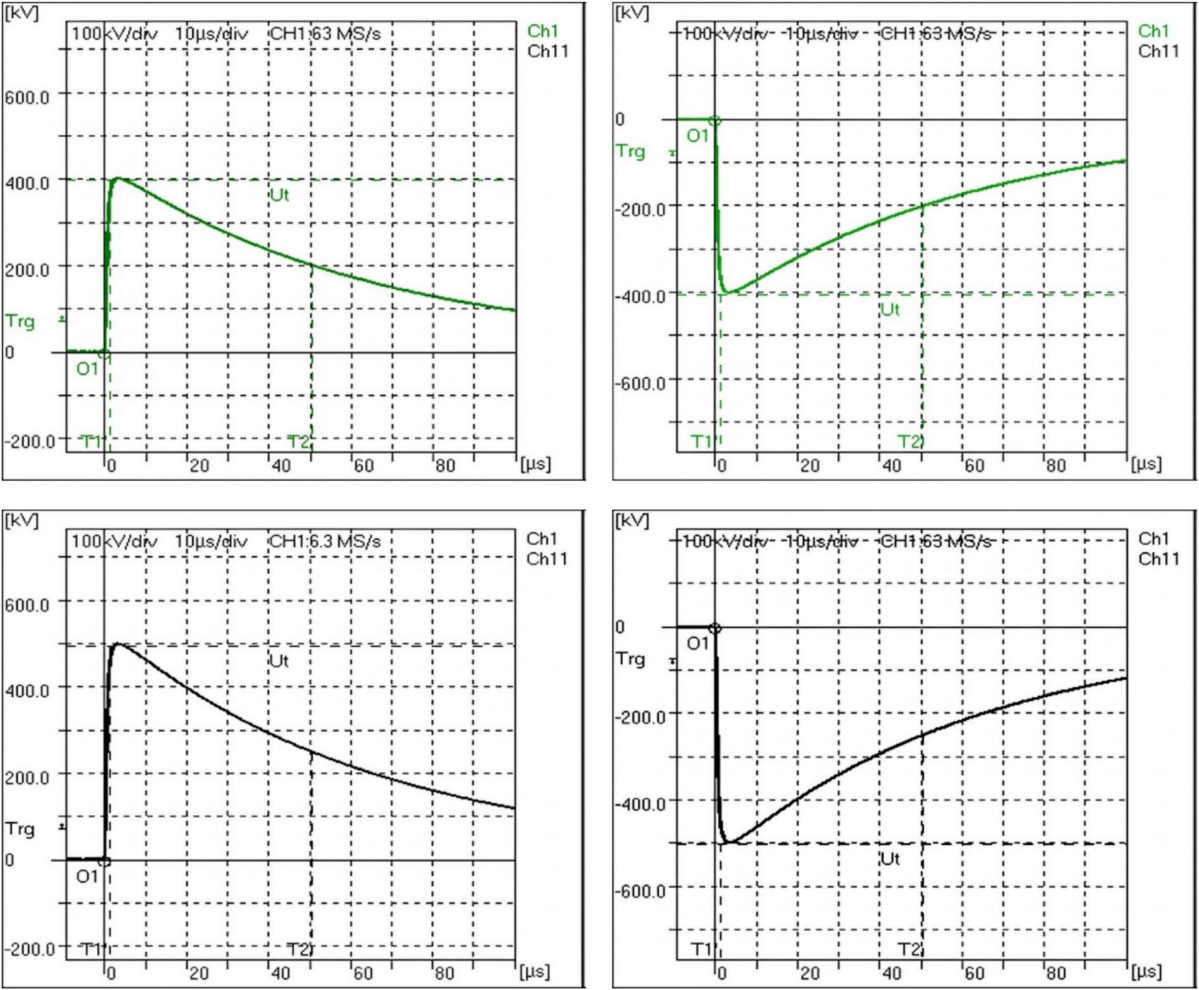
Impulse waveform measured by standard system for voltages from 100 to 500 kV at Positive and negative polarities.

According to the circuit diagram in Fig. 3, the standard impulse measuring system is connected in parallel with the voltage divider of the equipment under calibration. Also, the standard DC measuring system is connected to the DC voltage source for measuring the DC charging voltage. Five points are measured in the coverage area as 100 kV, 200 kV, 300 kV, 400 kV, and 500 kV peak in positive and negative polarities. For each calibration point, five measurements were recorded and the corresponding standard deviation was calculated to assess the repeatability of the measurements. Also, the mean value for each measurement was determined for impulse and DC voltages. Figure 4 shows waveforms of impulse voltage measured by standard system from 100 to 500 kV for positive and negative polarities. The measurement results are shown in Table 1. Measurement error (E) represents the accuracy of the calibration object and calculated by Eq. (3).

3 $$E(\% ) = \frac{{U_{{imp(uuc)}} - U_{{imp(Ref)}} }}{{U_{{imp(Ref)}} }} \times 100$$

where ${U}_{imp(ucc)}$ is the impulse measured voltage of the unit under calibration (kV); ${U}_{imp(Ref)}$ is the impulse measured voltage of the reference standard (kV).

**Table 1:** Impulse high voltage measurement results at a voltage range within 500 kV.

Polarity	Impulse measured value of UUC (kV)	Impulse measured value of standard (kV)	Measurement error (%)	DC measured value (kV)	Efficiency	Linearity error (%)
+ ve	99.45	98.994	0.46	13.18	0.942	− 0.085
	200.6	200.63	− 0.01	26.70	0.943	0.021
	301.8	301.73	0.02	40.12	0.943	0.021
	400.5	400.79	− 0.07	53.28	0.943	0.021
	498.1	498.73	− 0.13	66.29	0.943	0.021
− ve	99.67	99.607	0.06	13.27	0.942	− 0.064
	201.1	201.15	− 0.02	26.75	0.943	0.042
	301.0	301.01	− 0.003	40.02	0.943	0.042
	400.8	400.77	0.007	53.28	0.943	0.042
	498.7	498.71	− 0.002	66.32	0.942	− 0.064

It has been observed that, the measurement error values not exceed 3%. This indicates that the UUC falls within the specified ranges of accuracy.

**Fig. 5:**
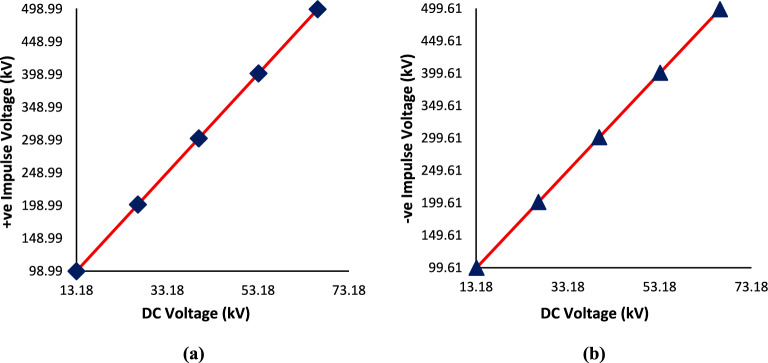
Relationship between the standard impulse and DC voltages at: (**a**) Positive polarity, (**b**) Negative polarity.

The average value of the efficiency factor represents the scale factor for the measurements beyond the standard range. Its value is equal 0.943 for both polarities at 8-stages. According to Table 1 and Fig. 5, it has been observed that linearity error in all calibration points is less than 1%. This means that the calculated efficiency is valid for making extension for high voltage measurements up to 800 kV. Therefore, the relationship between the standard impulse voltage and the DC voltage is as U_imp_ = 0.943 × 8 × U_dc_, according to Eq. (2).

Also, Tables 2 and 3 show the measured front and tail time during the calibration process at each voltage. A slight asymmetry between positive and negative polarity results is observed, particularly in the front time (T1). This behavior can be attributed to the polarity-dependent breakdown characteristics of spark gaps and differences in discharge development within the multistage impulse generator. In addition, measurement system factors such as stray capacitance and grounding configuration may contribute to this effect.

**Table 2:** Front time (T_1_) measurement results.

Polarity	Impulse measured value of standard (kV)	Front time measured value of UUC (μs)	Front time measured value of standard (μs)	Measurement error (%)
+ ve	98.994	1.28	1.269	0.87
	200.63	1.29	1.283	0.55
	301.73	1.30	1.278	1.72
	400.79	1.30	1.282	1.40
	498.73	1.28	1.279	0.08
− ve	99.607	1.28	1.255	1.99
	201.15	1.28	1.263	1.35
	301.01	1.29	1.257	2.63
	400.77	1.31	1.265	3.56
	498.71	1.31	1.264	3.64

**Table 3:** Tail time (T_2_) measurement results.

Polarity	Impulse Measured value of standard (kV)	Tail time measured value of UUC (μs)	Tail time measured value of standard (μs)	Measurement error (%)
+ ve	98.994	51.17	50.637	1.05
	200.63	51.53	50.660	1.72
	301.73	51.59	50.681	1.79
	400.79	51.62	50.767	1.68
	498.73	51.75	50.845	1.78
− ve	99.607	51.47	50.845	1.23
	201.15	51.58	50.839	1.46
	301.01	51.61	50.823	1.55
	400.77	51.59	50.911	1.33
	498.71	51.73	50.965	1.50

### Calibration of impulse voltage above the standard measurement range

In this stage, the standard impulse divider is disconnected and removed from the circuit for voltages above 500 kV due to its rated measurement limit. Operating the standard divider beyond its specified range may lead to potential damage to the equipment. Therefore, its removal is necessary to ensure safe and compliant operation according to standard practice.

On the other hand, the standard DC measuring system is still connected to the DC voltage source for measuring the DC charging voltage during all calibration points. By the calibration object, the output impulse voltage is generated at both polarities. Three points of impulse voltage are calibrated in this stage as 600 kV, 700 kV, and 800 kV. Each impulse voltage has been measured with its corresponding DC voltage. The measurement results are shown in Table 4.

**Table 4:** Measurement results of impulse voltages above 500 kV peak.

Polarity	Impulse Measured value of UUC (kV)	Standard calculated impulse value using efficiency factor (kV)	Measurement error (%)
+ ve	601.2	595.60	0.94
	699.4	693.44	0.86
	799.9	792.87	0.89
− ve	602.7	595.83	1.15
	701.5	693.90	1.10
	800.9	793.63	0.92

### Uncertainty estimation

Uncertainty budget is identified and assessed in accordance with Guide to the Expression of Uncertainty in Measurement-GUM^25^. The five single components that make up the uncertainty budget for this calibration are significant enough to be cleared in Table 5. Four of them are considered Type B uncertainties, and one is classified as Type A uncertainty. An illustration of uncertainty estimation for 300 kV calibration point is provided in Table 5 at positive polarity. Also, Table 6 displays the uncertainty values for each calibrated point up to 500 kV. It has been obtained that the percentage uncertainty of measurement is equal 0.87%. Therefore, 0.87% can be expressed as the overall uncertainty in measurements up to 500 kV range. Because of uncertainty values are less than 3%, the calibration object complies with the requirement of the related standard IEC 60060-2^24^ In addition, all factors of uncertainty budget are influencing in the calibration process.

**Table 5:** Uncertainty budget of impulse voltage measurement for 300 kV calibrated point.

Uncertainty sources	Standard uncertainty (kV)	Probability distribution, type	Divisor	Coefficient factor	Uncertainty contribution (kV)
Repeatability of the UUC readings	3.33 × 10^–2^	Normal, A	1	1	3.33 × 10^–2^
Calibration certificate of the standard	9.81 × 10^–1^	Normal, B	1	1	9.81 × 10^–1^
Resolution of the UUC	5.00 × 10^–2^	Rectangular, B	$\sqrt{3}$	1	2.89 × 10^–2^
Accuracy of the standard	15.1 × 10^–1^	Rectangular, B	$\sqrt{3}$	1	8.71 × 10^–1^
Short term stability of the measured voltage	1.00 × 10^–1^	Rectangular, B	$\sqrt{3}$	1	5.77 × 10^–2^
Combined standard uncertainty					1.31
Expanded uncertainty [at confidence Level 95% with coverage factor (k = 2)]					2.63

**Table 6:** Expanded uncertainty estimation of calibrated points up to 500 kV.

Polarity	Impulse Measured value of UUC (kV)	Impulse Measured value of standard (kV)	Expanded uncertainty	
			± kV	± %
+ ve	99.45	98.994	0.86	0.87
	200.6	200.63	1.77	0.87
	301.8	301.73	2.63	0.87
	400.5	400.79	3.49	0.87
	498.1	498.73	4.34	0.87
− ve	99.67	99.607	0.88	0.88
	201.1	201.15	1.75	0.87
	301.0	301.01	2.62	0.87
	400.8	400.77	3.49	0.87
	498.7	498.71	4.34	0.87

For clarity, the uncertainty budget has been presented with a clear distinction between Type A and Type B components. Type A uncertainty is primarily represented by the repeatability of the UUC readings, which reflects the variability observed when repeating measurements multiple times under the same conditions. Type B uncertainties include several contributions: the calibration certificate of the standard, which represents the uncertainty declared by the reference laboratory for the standard device; the accuracy of the standard, which reflects the intrinsic performance specifications of the device itself; the resolution of the UUC, which accounts for the finite resolution of the measuring instrument; and the short-term stability of the measured voltage, which captures small variations in the output voltage over the short duration of the measurements. All components are treated as uncorrelated in the combined standard uncertainty calculation according to the GUM framework.

In order to evaluate the overall uncertainty of the range above 500 kV, the uncertainty of the efficiency factor should be estimated. Table 7 shows the uncertainty budgets that could contribute in efficiency factor measurement. It has been obtained from this evaluation that the uncertainty value of efficiency factor is 1.10% at 300 kV calibrated point and is the same for all points at both polarities. So, it can be considered that 1.10% is the expanded uncertainty of efficiency factor measurement. After that, the uncertainty budgets in the range above 500 kV are summarized in Table 8 and estimated for 700 kV calibrated point. Therefore, 1.32% can be expressed as the overall uncertainty in measurements above 500 kV range. Table 9 shows the obtained reference impulse voltage values at 600 kV, 700 kV, and 800 kV with the corresponding estimated uncertainty.

**Table 7:** Uncertainty budget of efficiency factor measurement at 300 kV calibrated point.

Uncertainty sources	Standard uncertainty (%)	Probability distribution, type	Divisor	Coefficient factor	Uncertainty contribution (%)
Repeatability of the efficiency factor	1.76 × 10^–2^	Normal, A	1	1	1.76 × 10^–2^
Calibration certificate of the standard impulse divider	3.25 × 10^–1^	Normal, B	1	1	3.25 × 10^–1^
Calibration certificate of the standard DC divider	3.30 × 10^–1^	Normal, B	1	1	3.30 × 10^–1^
Accuracy of the standard impulse divider	5.00 × 10^–1^	Rectangular, B	$\sqrt{3}$	1	2.89 × 10^–1^
Drift due to the DC divider	1.25 × 10^–2^	Rectangular, B	$\sqrt{3}$	1	7.22 × 10^–3^
Combined standard uncertainty					0.55
Expanded uncertainty [at confidence Level 95% with coverage factor (k = 2)]					1.10

**Table 8:** Uncertainty budget of high voltage measurement above 500 kV at 700 kV calibrated point.

Uncertainty sources	Standard uncertainty (%)	Probability distribution, type	Divisor	Coefficient factor	Uncertainty contribution (%)
Repeatability of the UUC	7.34 × 10^–2^	Normal, A	1	1	7.34 × 10^–2^
Calibration certificate of the standard DC divider	3.30 × 10^–1^	Normal, B	1	1	3.30 × 10^–1^
Efficiency factor	5.51 × 10^–1^	Normal, B	1	0.943	5.51 × 10^–1^
Resolution of the UUC	7.15 × 10^–3^	Rectangular, B	$\sqrt{3}$	1	4.13 × 10^–3^
Short term stability of the measured voltage	2.43 × 10^–1^	Rectangular, B	$\sqrt{3}$	1	1.40 × 10^–1^
Combined standard uncertainty					0.66
Expanded uncertainty [at confidence Level 95% with coverage factor (k = 2)]					1.32

**Table 9:** Expanded uncertainty estimation of calibrated points above 500 kV.

Polarity	Impulse measured value of UUC (kV)	Standard calculated impulse value (kV)	Expanded uncertainty ± (%)
+ ve	601.2	595.60	1.32
	699.4	693.44	
	799.9	792.87	
− ve	602.7	595.83	1.32
	701.5	693.90	
	800.9	793.63	

For all calibration points above 500 kV, a single expanded uncertainty value of 1.32% is assigned as a conservative worst-case estimate. This approach ensures that potential variations in the efficiency factor at higher voltages are accounted for, providing a reliable representation of measurement uncertainty.

## Validation of results

Verifying the obtained results is crucial to guaranteeing the precision and reliability of the suggested methodology. The UUC has a calibration certificate included in its manual and it was carried out by HIGHVOLT Calibration Laboratory for Electrical Measuring Quantities and High Voltage Measuring Systems at Germany. The outcomes of this certificate could be used to be compared with the results of the current proposed method as shown in Table 10. The normalized error tool (E_n_) has been used as an evaluation criterion according to Eq. (4)^26^.

4 $$E_{n} = \frac{{\left| {X_{1} - X_{2} } \right|}}{{\sqrt {U_{1}^{2} + U_{2}^{2} } }}$$

where X_1_ and X_2_ are the deviation error value of the proposed method and the HIGHVOLT Calibration Laboratory respectively in kV. U_1_ and U_2_ are expanded uncertainty obtained from the proposed method and the HIGHVOLT Calibration Laboratory respectively in kV.

**Table 10:** Comparison between the suggested method and the HIGHVOLT Calibration Laboratory.

Polarity	Nominal value (kV)	Suggested scale factor method		HIGHVOLT calibration laboratory		Normalized error (E_n_)
		Error (kV)	Expanded uncertainty ± (%)	Error (kV)	Expanded uncertainty ± (%)	
+ ve	600	5.60	1.32	13.26	1.70	0.593
	800	7.03		11.31		0.248
− ve	600	6.87	1.32	10.69	1.70	0.296
	800	7.27		8.80		0.089

The interpretation of E_n_ is as follows:

- When E_n_ is less than or equal to 1, it indicates satisfactory results.
- When E_n_ is greater than 1, it indicates unsatisfactory results.

**Fig. 6:**
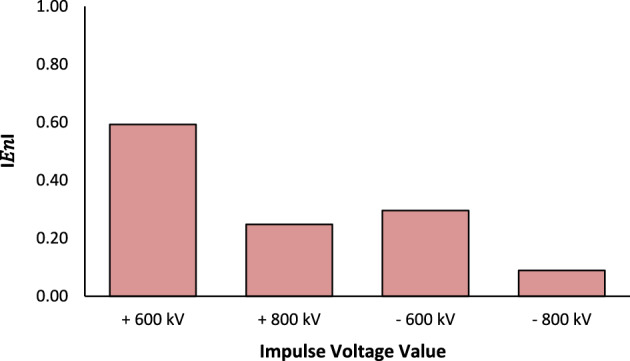
Normalized error of impulse voltage measurement.

**Fig. 7:**
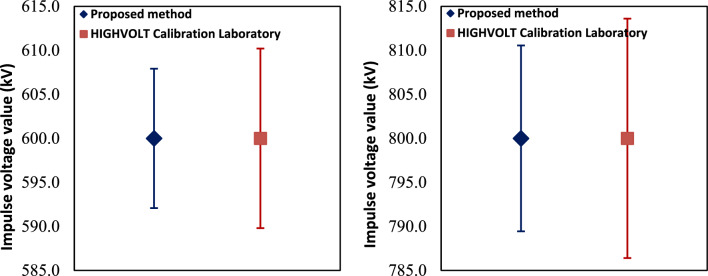
Comparison between proposed method and the UUC calibration certificate at 600 kV and 800 kV.

According to Table 10 and Figs. 6 and 7, it has been noticed that a slightly higher normalized error is observed for positive polarity compared to negative polarity. This behavior may be attributed to minor asymmetries in the impulse generation process, including differences in spark gap breakdown characteristics and discharge dynamics, as well as small measurement system effects such as grounding configuration and stray capacitance. Nevertheless, all E_n_ values remain below 1.0, confirming that the calibration procedure is reliable and the proposed efficiency factor method accurately extends the calibration range up to 800 kV.

## Limitations and future work

- While the efficiency factor method demonstrates linearity errors below 1% up to 800 kV under controlled conditions (23 ± 2 °C, 50 ± 10% RH), it is important to note that physical factors—such as spark gap characteristics, stray capacitance, corona loss, and generator stage efficiency—may affect the linearity under extreme or varying conditions. Therefore, it is recommended to monitor the DC charging voltage during each calibration and to periodically verify the efficiency factor against a reference standard to ensure continued reliability. Future work should focus on investigating the variation of the efficiency factor under different temperatures, humidity levels, long-term aging, and multiple calibration cycles, in order to refine the method and enhance its applicability for extended service periods.
- It should be noted that the uncertainty of the efficiency factor, evaluated from measurements within the 500 kV range, was applied to estimate the uncertainty for the extended voltage range (600–800 kV). While this approach assumes that the efficiency factor remains stable, it may not fully capture additional contributions introduced by extrapolation. All sub-components of the efficiency factor uncertainty were treated as uncorrelated for simplicity. However, the conservative estimation, combined with normalized error verification (E_n_ < 1.0), indicates that the extended voltage uncertainties remain within acceptable limits. Future work may involve more detailed modeling of potential correlations and additional experiments at higher voltages to further refine the uncertainty evaluation.
- The validation of the proposed method using the original calibration certificate of the UUC provides a practical consistency check; however, it has inherent limitations due to possible performance drift of the system over time. Additional independent validation methods, such as inter-laboratory comparisons, the use of higher-range reference standards, or alternative measurement techniques, would further strengthen the reliability of the method for voltages exceeding 500 kV. These aspects represent important directions for future work.

## Conclusion

The primary aim of this research is to tackle the calibration challenge faced when calibrating impulse high-voltage measuring devices on-site due to the absence of reference devices within the same voltage range. As technology progresses, impulse high-voltage testing systems now boast significantly high ranges, surpassing the capabilities of available portable calibration devices. To address this hurdle, this paper introduces a specialized calibration method tailored for impulse high-voltage testing systems operating within an 800 kV range.

This proposed technique relies on using a standard divider with a 500 kV peak range for impulse voltage measurement and another for DC voltage with a range of 100 kV. To extend the calibration scope beyond 500 kV, an efficiency factor method is introduced. Results derived from the calibration procedure demonstrate linearity across all calibrated points, with a linearity error of under 1%, affirming the reliable extension of calibration up to 800 kV. The study also verifies that the unit undergoing calibration aligns with the accuracy standards specified in its documentation.

Moreover, the research assesses the expanded uncertainty for each calibration range, offering a comprehensive insight into the measurement uncertainties associated with the calibration process. Introducing a comparison and verification tool utilizing a normalized error metric (E_n_) ensures the reliability of the results. The outcomes from the normalized error analysis validate the effectiveness of the proposed calibration method, ensuring the satisfactory nature of the obtained results.

Essentially, this paper facilitates the provision of calibration services for impulse high-voltage testing systems featuring significantly broad voltage ranges directly at the customer’s site, addressing the existing challenges in the field of high-voltage calibration.

Future work will focus on extending the experimental verification to a wider range of environmental conditions, multiple calibration cycles, independent validation at high voltages, and refinement of the uncertainty model to account for potential correlations and nonlinear effects.

## Data Availability

The data sets used and/or analyzed during the current study are available from the corresponding author on reasonable request.
